# Mechanisms of action of adrenocorticotropic hormone and other melanocortins relevant to the clinical management of patients with multiple sclerosis

**DOI:** 10.1177/1352458512458844

**Published:** 2013-02

**Authors:** Barry G Arnason, Regina Berkovich, Anna Catania, Robert P Lisak, Mone Zaidi

**Affiliations:** 1 Department of Neurology, University of Chicago Medical Center, USA; 2 Department of Neurology, University of Southern California Keck School of Medicine, USA; 3 Center for Preclinical Investigation, Fondazione IRRCS Ca’ Granda-Ospedale Maggiore Policlinico, Italy; 4 Department of Neurology, Wayne State University, USA; 5 The Mount Sinai Bone Program, Mount Sinai School of Medicine, USA

**Keywords:** Multiple sclerosis, melanocortins, melanocortin signaling, ACTH, corticosteroids

## Abstract

The therapeutic benefits of adrenocorticotropic hormone in multiple sclerosis are usually ascribed to its corticotropic actions. Evidence is presented that adrenocorticotropic hormone, approved for multiple sclerosis relapses, acts via corticosteroid-independent melanocortin pathways to engender down-modulating actions on immune-system cells and the cytokines they synthesize. Immune response-dampening effects are also brought about by agent-induced neurotransmitters that inhibit immunocytes. The likelihood that adrenocorticotropic hormone promotes microglial quiescence and counteracts glucocorticoid-mediated bone resorption is discussed.

## Introduction

Adrenocorticotropic hormone (ACTH) is cleaved from its pro-opiomelanocortin (POMC) prohormone as are α-melanocyte-stimulating hormone (α-MSH), β-MSH and γ-MSH (Figure 1a).^1^ ACTH and α-, β- and γ-MSH comprise the melanocortins. There are five melanocortin receptors (MCRs). ACTH binds to all MCRs, with only MC2R implicated in adrenal steroidogenesis.^1^ ACTH gel (H.P. Acthar^®^ Gel; Questcor Pharmaceuticals Inc, Hayward, CA, USA), a slow-release formulation of full-sequence ACTH_(1–39)_ (80 units/ml), contains additional biologically active POMC peptides (personal communication, David Young). ACTH gel has been used for decades to treat multiple sclerosis (MS) exacerbations. In 2010, the US Food and Drug Administration re-approved ACTH gel for treatment of acute exacerbations of MS in adults after a re-examination of the study data submitted in 1978. These early studies demonstrated that ACTH gel led to faster recovery than placebo in rapidly worsening MS.^2,3^ They also antedated an appreciation of the full physiologic actions of melanocortins.

**Figure 1a.**
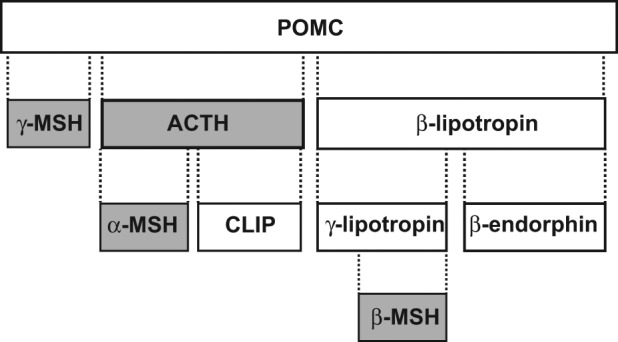
Melanocortin peptides (shaded boxes) derived from POMC. ACTH: adrenocorticotropic hormone; CLIP: corticotropin-like intermediate lobe peptide; MSH: melanocyte-stimulating hormone; POMC: pro-opiomelanocortin, Reprinted with permission from: Catania A, et al. *Pharmacol Rev* 2004; 56: 1-29.^1^

Before MCRs were discovered, ACTH efficacy was thought to rest solely on its corticotropism. This presumption, plus the efficacy of corticosteroids, played a role in the acceptance of high-dose corticosteroids as the preferred treatment for MS exacerbations. Recent data in nephrotic syndrome,^4^ opsoclonus-myoclonus^5^ and infantile spasms (IS)^6^ indicate that steroidogenesis cannot explain why ACTH benefits these conditions. The benefit of ACTH in IS, a brain disease, indicates that systemic ACTH improves brain function. Improvement cannot be glucocorticoid-mediated, because glucocorticoids provide doubtful benefit in IS,^6^ and efficacious ACTH doses far exceed doses that maximally increase glucocorticoid release. Similarly, ACTH has benefitted patients with nephrotic syndrome^4^ and opsoclonus- myoclonus^5^ who failed corticosteroid therapy. Given these observations, the credo that the benefit of ACTH in MS rests on steroidogenesis merits reconsideration. This review considers mechanisms that likely underlie differences in efficacy and tolerability/safety between ACTH and glucocorticoids.

POMC is cleaved to form ACTH_(1–39)_ and other peptides. ACTH can, in turn, be cleaved to form α-MSH (Figure 1a).^1^ The POMC-melanocortin host-defense system has been conserved for 500 million years. Its glucocorticoid-independent functions include: control of melanogenesis, food intake, energy expenditure and sexual function.^1^ Melanocortins also improve attention, memory and learning, and affect behavior.^7^ The corticosteroid- independent anti-inflammatory effects of melanocortins germane to MS are the focus of this review. We also compare ACTH and corticosteroids with respect to safety and tolerability.

## MCR distributions and functions

MC1R was the first melanocortin receptor cloned;^8^ cloning of four additional MCRs followed promptly. MCRs are found in multiple cell types, but even today knowledge of the extent of their expression remains incomplete. MC1R is expressed in skin in melanocytes and in monocytes, neutrophils and lymphocytes.^1^ MC1R is also expressed in central nervous system (CNS) microglia and astrocytes.^9^ MC2R, the adrenal receptor underlying the steroidogenic actions of ACTH,^1^ is also found in osteoblasts^10^ and skin.^9^ MC3R is found in the hypothalamus and limbic system,^1^ whereas MC4R, the dominant CNS receptor, is expressed in the cortex, thalamus, hypothalamus, brain stem and spinal cord.^11^ MC5R, widely distributed in exocrine glands, is also found in lymphocytes.^1^

Melanocortins are anti-inflammatory at cellular (i.e. directly suppressing immunocytes) and system levels (i.e. nervous system and glucocorticoid-mediated immune-system downregulation). MC1R, the dominant receptor in immunocytes, is expressed by all cell types that respond to melanocortin-mediated anti-inflammatory signals including monocytes, macrophages, microglia, neutrophils, mast cells and astrocytes.^9^ MC5R can shift T-cell phenotype from immune activating to tolerogenic.^1^ Engagement of monocyte/macrophage MC3Rs is likewise anti-inflammatory.^1^ The distributions and roles of the various MCRs are summarized in Table 1.^1,9^

**Table 1.** Distribution and functions of melanocortin receptors (MCRs).

MCR subtype	Affinity	Organs	Cell types	Functions
MC1R	α-MSH = ACTH >> γ-MSH	Brain, skin, gut, testis, ovary, placenta, lung, liver, adrenal	Microglia, monocytes/ macrophages, lymphocytes, neutrophils, astrocytes, melanocytes, keratinocytes, fibroblasts, endothelium, microvascular endothelium, intestinal epithelia, Leydig cells, lutein cells, trophoblasts	Antipyretic effects Pigmentary effects Anti-inflammatory effects
MC2R	ACTH	Adrenal, testis, skin, adipose tissue, pancreas, bone^a^	Zona fasciculata and glomerulosa cells, adipocytes, keratinocytes, pancreatic islet β-cells, osteoblasts^a^	Steroidogenesis Bone protection^a^
MC3R	γ-MSH = ACTH ≥ α-MSH	Brain, heart, immune system, skeletal muscle	Macrophages, monocytes, lymphocytes, neurons	Autonomic functions Anti-inflammatory effects Neuroprotection Control of feeding/energy Erectile activity NA and ACh-mediated anti-inflammatory effects
MC4R	α-MSH = ACTH >> γ-MSH	Brain/central nervous system, skin, skeletal muscle	Dermal papilla, skeletal myocytes, neurons, astrocytes	
MC5R	α-MSH ≥ ACTH > γ-MSH	Brain, spleen, bone marrow, skeletal muscle, skin, exocrine glands, lung, heart, kidney, adipose tissue, adrenal, uterus, ovary	Macrophages, lymphocytes, adipocytes, skeletal myocytes, intestinal epithelium	• Immunoregulation, exocrine secretion

a Zaidi M, et al. *Proc Natl Acad Sci U S A* 2010; 107: 8782–8787.

ACTH: adrenocorticotropic hormone; MSH: melanocyte-stimulating hormone.

Adapted with permission from: Catania A, et al. *Pharmacol Rev* 2004; 56: 1–29; Brzoska T, et al. *Endocr Rev* 2008; 29: 581–602.^1,9^

## Ligands for MCRs

All melanocortins share the histidine, phenylalanine, arginine, tryptophan (HFRW) core sequence.^1^ Shared HFRW explains why all melanocortins bind to every MCR except MC2R. The Lys-Lys-Arg-Arg (KKRR)_(15–18)_ motif, the address sequence that permits MC2R recognition, is unique to ACTH^1^ and explains why ACTH is the primary melanocortin that binds to MC2Rs.

Affinities of the melanocortins for receptors differ. ACTH and α-MSH recognize MC1R and MC4R with equal affinities, whereas α-MSH binds preferentially to MC5R and γ-MSH to MC3R (Table 1).^1,9^ Most melanocortin studies use α-MSH, which precludes confusion with glucocorticoid actions. Still, α-MSH effects extrapolate to ACTH, given their shared affinities for MCRs.

## Direct immunomodulatory effects of melanocortins

Melanocortins inhibit innate immune system cells widely, including macrophages peripherally and microglia in the CNS.^9^ Stimulation of brain-expressed MC4Rs increases central release of neurotransmitters (e.g. noradrenalin (NA), acetylcholine (ACh), dopamine (DA)) that can quell microglia and, via descending neural pathways, triggers release in the periphery of anti-inflammatory NA and ACh from sympathetic and parasympathetic nerve endings.^7,11,12^

α-MSH restrains the nuclear factor-kappaB (NF-κB) transcription factor.^9,13,14^ NF-κB remains inactive in resting cells, bound in the cytoplasm to IκB inhibitory protein family members. Phosphorylation of IκB by cytokines, bacterial products, or viruses causes IκB degradation. Newly freed NF-κB translocates to the nucleus and binds to DNA sequences encoding NF-κB-responsive elements that then trigger transcription of target genes.^13^ Melanocortins prevent this by generating cyclic adenosine monophosphate (cAMP), thereby blocking IκBα phosphorylation and NF-κB translocation (Figure 1b).^13^ α-MSH-inhibited, NF-κB-controlled, proinflammatory mediators include interleukin (IL)-1, IL-6, IL-8 and tumor-necrosis factor (TNF)-α.^9^ NF-κB is a master proinflammatory switch. In accordance, MCR engagement has broad immunoinhibitory consequences.

**Figure 1b.**
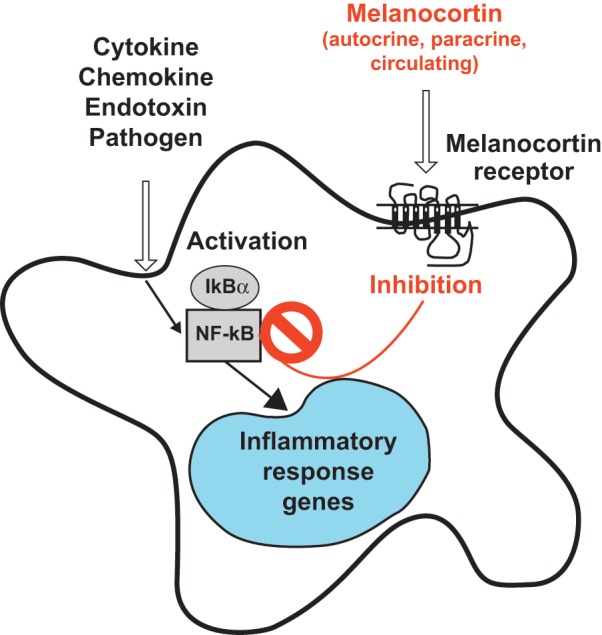
Melanocortin inhibition of NF-kB activation in immunocytes. NF-lB is inactive in the cytoplasm, bound to IlB. Cytokines, chemokines, endotoxins and pathogens cause IlB phosphorylation and degradation. Free NF-lB translocates to the nucleus to trigger transcription of proinflammatory molecules. MCR activation by melanocortins inhibits IlBa phosphorylation and NF-lB nuclear translocation. MCR: melanocortin receptor; NF-lB: nuclear factor-kappaB. Reprinted with permission from: Catania A. *J Leukoc Biol* 2007; 81: 383–392.^13^

Melanocortins inhibit adhesion molecule expression and IL-8-mediated neutrophil chemotaxis. Both actions impede immunocyte entry into the CNS.^9^ Tissue-destructive nitric oxide and neopterin release from macrophages (which rises during MS flares^15^) is also reduced by melanocortins.^9^

α-MSH increases production of anti-inflammatory IL-10. This reinforces the immunosuppressive capacity of α-MSH.^1^ Interestingly, IL-10 levels are subnormal in progressive MS.^16^ α-MSH and ACTH induce regulatory T cells (Tregs) that suppress disease in the experimental autoimmune encephalomyelitis (EAE) model of MS.^17^ In experimental autoimmune uveitis, α-MSH helps convert primed CD4^+^ T cells into CD4^+^, CD25^+^ Tregs that suppress disease; conversion is driven by MC5R binding.^1^ Perhaps of relevance, CD8^+^ Tregs function minimally during MS attacks.^18^

## CNS-mediated anti-inflammatory effects

Animal studies using intracerebroventricular melanocortins^1^ point to multiple CNS POMC^+^ neuron-initiated and CNS-confined effects. Because systemic ACTH induces such effects, the agent must somehow activate CNS-restricted POMC^+^ neurons.^7^

POMC^+^ neurons are located in the hypothalamic arcuate nucleus (ARC) and in the medullary nucleus tractus solitarius (NTS).^11^ The NTS abuts the area postrema, which lacks a blood-brain barrier, as do the medial NTS and medial ARC.^19,20^ Systemically administered ACTH thus has access to neural elements in these loci. POMC^+^ ARC neurons express MC3Rs and MC4Rs; NTS neurons, MC4Rs.^21^ Systemic α-MSH/ACTH accumulated along the margins of the area postrema can spread into neighboring brain parenchyma. Central terminals of vagal afferents that express MC4Rs synapse onto POMC^+^ neurons of the NTS.^21^ Locally accumulated α-MSH/ACTH binds to the MC4Rs and glutamate release follows.^21^ These same vagal afferent neurons likewise express MC4Rs on their opposite nerve endings in the gut.^22^ Systemic ACTH has ready access to these receptors. ACTH binding to MC4Rs potentiates afferent vagal firing, and provokes additional glutamate release onto POMC^+^ NTS neurons.^21^ Glutamate, released in response to vagus-mediated signals, is thought to increase NTS POMC synthesis, POMC processing and melanocortin release from POMC^+^ NTS neuron axon terminals expressed widely in the brain stem and beyond.

POMC^+^ ARC neurons send descending axons to the dorsal motor vagal nucleus (DMVN).^11^ α-MSH/ACTH released from axon terminals binds to MC4Rs, abundant on DMVN neurons.^11^ Engagement provokes firing and ACh release from distal vagal efferent nerve endings.^23^ These endings also express MC4Rs,^22^ to which systemic ACTH has ready access and ACTH binding to them triggers additional distal ACh release.

Descending axons carry α-MSH/ACTH from POMC^+^ ARC neurons to the dorsolateral thoracic spinal cord.^11^ α-MSH/ACTH, when released, binds to MC4Rs on spinal cord preganglionic sympathetic nervous system (SNS) neurons.^11^ Preganglionic neurons relay onto paravertebral SNS ganglion neurons that then release NA at multiple distal sites including the lymphoid organs. In addition, ACTH increases tyrosine hydroxylase mRNA, and hence NA synthesis, in SNS ganglion neurons.^24^ ACTH bolsters these anti-inflammatory effects by binding directly to MCRs expressed on distal SNS axons; distal binding triggers additional NA release.^25^

### Modulation of inflammation by NA

NA released from SNS nerve endings binds to β_2_-adrenergic receptors on monocytes/macrophages.^26^ Binding transduces cAMP-mediated signals that reduce production of proinflammatory cytokines, including TNF-α.^26^ NA protects against EAE,^26^ suggesting that melanocortin-induced NA release may be relevant in MS, a notion reinforced by findings with the β_2_-adrenergic agonist albuterol which, as an add-on to glatiramer acetate, strikingly reduces MS relapses.^27^

### Modulation of inflammation by ACh

Peripheral cholinergic pathways downregulate inflammatory responses via macrophage-expressed α7-nicotinic ACh receptors (AChRs).^23^ DMVN neuron activation by central α-MSH/ACTH triggers ACh release from their efferent endings.^23^ Released ACh engages monocyte/macrophage α7-nicotinic AChRs. Engagement activates cAMP that blocks NF-κB nuclear translocation, inhibits proinflammatory cytokine synthesis and promotes anti-inflammatory cytokine release by monocytes/macrophages.^23^

Beyond this, vagal efferent endings synapse on α7-nicotinic AChR-expressing SNS cell bodies in celiac ganglia.^28^ ACh binding to these endings activates celiac ganglion SNS neurons; activation is followed by augmented distal NA release in the spleen.^28^ Thus, melanocortins increase immunosuppressive NA outflow via four reinforcing mechanisms.

### CNS-restricted effects of melanocortins

Systemic ACTH (or α-MSH) heightens motivation, attention, arousal, learning and memory.^7^ These CNS-restricted actions establish that ACTH (or α-MSH) given peripherally, but acting centrally, favorably modulates CNS-restricted neurotransmission. Specifically, MCR signaling potentiates CNS-restricted NA release from locus ceruleus (LC) nerve endings.^29^ MCR signaling also increases tyrosine hydroxylase mRNA and, hence, NA synthesis within the LC.^29^ Released NA, bound to β2-adrenegic receptors on microglia, promotes microglial quiescence.^12^ To the contrary, LC ablation causes NA depletion throughout the cortex and frees microglia to make proinflammatory cytokines.^30^ Ultimately, decreased LC metabolism in MS links to cognitive impairment.^31^

MCR signaling increases CNS-restricted ACh and dopamine release.^7^ Microglia express tranquility-promoting α7-nicotinic ACh and dopamine receptors; additional quiescence-promoting receptors include gamma-aminobutyric acid (GABA) receptors^12^ and some, albeit not all, serotonin receptors.^32^ Glutamate, in contrast, activates microglia.^12^ Thus, several neurotransmitters conjointly counteract glutamate to promote microglial restraint. When inhibitory neurotransmitter signaling fails, microglial over activation will likely ensue. In progressive MS, microglia are activated globally,^33^ perhaps because of the loss of restraining (i.e. NA, ACh, DA, serotonin and GABA) in later-stage disease. Melanocortins might attenuate such deficiencies.

### Direct melanocortin actions on microglia

In MS, CNS-restricted proinflammatory cytokines (e.g. TNF-α) made by microglia are upregulated, whereas anti-inflammatory cytokines (e.g. IL-10) are suppressed.^16^ Melanocortins bound to MC1Rs on activated microglia suppress production of the proinflammatory mediators TNF-α, IL-6 and nitric oxide.^1,9^ These findings suggest yet another possible disease-ameliorating role for ACTH in MS.

In summary, ACTH is potently anti-inflammatory. ACTH binding to central and peripheral MCRs downregulates immunocyte activity, an effect superimposed onto its better known glucocorticoid-mediated actions (Figure 1c). In addition, ACTH potentiates parasympathetic and SNS activity. Both lessen immune responsiveness.

**Figure 1c.**
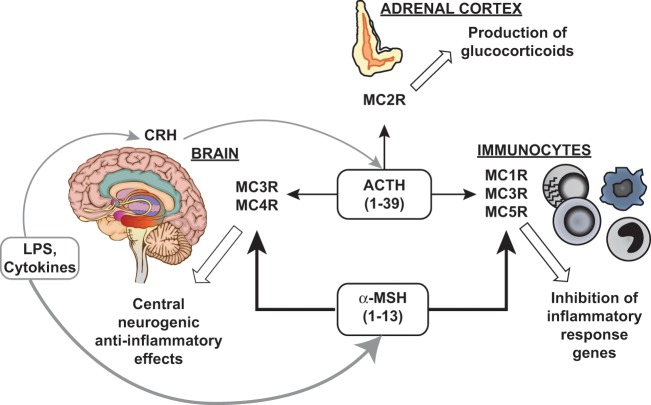
ACTH/melanocortin anti-inflammatory circuits. Pathogens, endotoxins (LPS), cytokines and other stressors enhance hypothalamic secretion of corticotropin-releasing hormone (CRH), which induces POMC processing and ACTH release from anterior pituitary. Circulating ACTH activates adrenal MC2Rs and promotes a systemic glucocorticoid-mediated anti-inflammatory response. Additionally, ACTH activates MCR subtypes within the brain (MC3R and MC4R) and elsewhere (MC1R, MC3R and MC5R). ACTH exerts glucocorticoid-independent anti-inflammatory actions at sites of inflammation and via central neurogenic pathways. LPS and cytokines induce POMC processing in extrapituitary cells, including brain cells, immunocytes, endothelial cells and keratinocytes (not discussed herein). The main product is α-MSH that likewise exerts potent anti-inflammatory actions. ACTH: adrenocorticotropic hormone; LPS: lipopolysaccharide; MCR: melanocortin receptor; MSH: melanocyte-stimulating hormone; POMC: pro-opiomelanocortin. Reprinted with permission from: Catania A. *J Leukoc Biol* 2007; 81: 383–392.^13^

## Melanocortin signaling and drug safety

Drug safety is a constant concern. Complications may surface long after a drug is approved. ACTH, used for 60 years to treat thousands of patients, has an established safety profile. Because ACTH has been linked to corticotropic effects, many assume that side effects of ACTH reflect those associated with corticosteroids. Yet, during ACTH treatment of MS, peak steroid levels are elevated trivially compared with those that follow megadose intravenous (IV) steroids. Besser et al.^34^ showed plasma 11-hydroxycorticosteroid levels of 69 μg/100 mL following intramuscular (IM) administration of 80 units of ACTH gel, whereas Coburg et al.^35^ observed peak plasma 17-hydroxycorticosteroid levels above 1500 μg/100 mL following IV administration of 1 g of prednisolone (~800 mg methylprednisolone). A 20-fold difference in peak steroid levels, given comparable potency, indicates that IV steroid and ACTH treatments differ substantially.

### Osteoporosis and avascular necrosis

Safety concerns with corticosteroids may be misapplied to ACTH. Osteoporosis and avascular necrosis provide examples.

High-dose corticosteroids are a leading cause of bone loss.^36^ Skeletal effects are dose- and duration-dependent. Acute effects of high-dose steroids implicate osteoblasts (i.e. bone-forming cells), which cease functioning and die by apoptosis. Prolonged steroid use reduces estrogen, testosterone, renal androgens and gastrointestinal calcium absorption while increasing calcium excretion and parathormone.^36^ All contribute to excessive bone removal.^36^ Steroids also induce osteonecrosis purportedly via increased osteoblast apoptosis.^36^

Animal studies support a role for ACTH in bone protection and formation. ACTH, contrary to steroids, promotes osteoblast differentiation from an immature into a mineralizing phenotype and counteracts dexamethasone-induced osteoblast apoptosis. ACTH stimulation of MC2R on osteoblasts correlates with elevated vascular endothelial growth factor (VEGF), an osteoblast activator.^10^ ACTH_(1–24)_-stimulated VEGF production reduces corticosteroid-induced osteonecrosis.^10^

Clinical observations similarly support an osteoprotective role for ACTH. Noted are lower bone loss in Cushing’s disease versus patients with adrenal adenomas, and higher bone mass in familial glucocorticoid deficiency (with elevated ACTH) versus age-matched controls.^10^

## Clinical significance and future directions

Clinical trials have demonstrated similar group efficacies for ACTH and IV steroids in MS relapses.^37^ A three-day course of IV treatment has advantages over a protracted course of IM injections in terms of patient comfort and medical resources,^37^ although these advantages might be offset should ACTH be shown to heighten motivation, arousal, attention, memory and learning in MS patients as it does in healthy individuals. Likewise, some patients who cannot tolerate steroids will tolerate ACTH. These differences would be surprising if ACTH effects depended solely on increased steroid release. Also worth considering are possible safety advantages of ACTH, including a potential reduced risk of bone loss, a problem for many on long-term or frequent pulse glucocorticoid therapy.

Despite extensive preclinical data on ACTH and melanocortins, questions linger. Ongoing studies of immunologic aspects of ACTH may further understanding of its mechanism(s) of action in treating MS. In addition, ACTH pulse therapy in relapsing–remitting or secondary progressive MS might prove informative. Whether ACTH-triggered CNS-restricted neurotransmitter release (e.g. NA, ACh and DA) can favorably affect progressive MS merits consideration. Basic science may clarify the effects of ACTH on bone. Also desired are trials that evaluate ACTH effects on bone in patients using steroids frequently.

## Conclusions

Study of the effects of melanocortins on immune function and inflammatory processes indicates that actions of ACTH in MS go beyond corticotropic effects. Additional research to pinpoint those melanocortin-driven actions of ACTH most relevant to MS may guide treatment decisions and improve understanding of the disease process.
